# Supplementary material for: Nucleosome wrapping energy in CpG islands and the role of epigenetic base modifications
Source: eLife. 2025 Dec 24;13:RP98468. doi: 10.7554/eLife.98468 (PMC12736940; doi:10.7554/eLife.98468)
Supplement: Supplementary file 1. [file elife-98468-supp1.pdf]

Supplementary information for the article "Nucleosome wrapping energy in CpG islands and the role of epigenetic base modifications"

Rasa Giniūnaitė [1,2], Rahul Sharma [3], John H. Maddocks [3],
Skirmantas Kriaučionis [4] and Daiva Petkevičiūtė-Gerlach [1,*]
[1] *Department of Applied Mathematics, Kaunas University of Technology, Studentų 50-318, 51368, Kaunas, Lithuania*
[2] *Institute of Mathematics, Vilnius University, Naugarduko 24, 03225, Vilnius, Lithuania*
[3] *Institut de Mathématiques, École Polytechnique Fédérale de Lausanne, EPFL SB MATH LCVMM Station 8, CH-1015 Lausanne Switzerland*
[4] *Ludwig Institute for Cancer Research Ltd, University of Oxford, Nuffield Department of Medicine, Old Road Campus Research Building, Roosevelt Drive, Oxford OX3 7DQ,UK*

i	CpG count interval	Mean score, NMI	Mean score, *not* NMI	NMI vs *not* NMI		i vs $i+1$, NMI		i vs $i+1$, *not* NMI	
				Δ	p-value	Δ	p-value	Δ	p-value
1	[0,4]	6.16	5.45	0.71	0.0001	1.47	0.0001	1.14	0.0001
2	[5,14]	4.69	4.31	0.38	0.0001	2.66	0.0001	2.43	0.0001
3	[15,24]	2.03	1.87	0.15	0.0001	1.53	0.0001	1.71	0.0002
4	[25,34]	0.50	0.16	0.33	0.0506	-	-	-	-

TABLE A: Nucleosome occupancy scores from Schwartz et al. [1], grouped by the genomic regions (NMI and not NMIs) and by indicated ranges of numbers of CpG dinucleotide steps, as in Figure 7a in the main text. Δ denotes the difference between mean scores, and p-values correspond to permutation tests for mean differences, applied due to non-normality of the data. Due to large sample sizes (see Table 2 in the main text), all but one of the mean differences are statistically significant.

i	CpG count interval	Mean score, NMI	Mean score, *not* NMI	NMI vs *not* NMI		i vs $i+1$, NMI		i vs $i+1$, *not* NMI	
				Δ	p-value	Δ	p-value	Δ	p-value
1	[0,4]	177.60	147.15	30.45	0.0001	-3.24	0.0608	-90.36	0.0001
2	[5,14]	180.84	237.51	-56.67	0.0001	66.68	0.0001	59.01	0.0001
3	[15,24]	114.16	178.50	-64.34	0.0001	94.91	0.0001	168.28	0.0001
4	[25,34]	19.25	10.22	9.03	0.0526	-	-	-	-

TABLE B: Nucleosome occupancy scores from Yazdi et al. [2], grouped by the genomic regions (NMI and not NMIs) and by indicated ranges of numbers of CpG dinucleotide steps, as in Figure 7b in the main text. See also the caption of Table A.

* To whom correspondence should be addressed. Email: daiva.petkeviciute@ktu.lt

Region	Mean score	CGI and NMI		CGI *not* NMI		*Not* CGI and NMI	
		Δ	*p*-value	Δ	*p*-value	Δ	*p*-value
CGI and NMI	2.60	0	-				
CGI *not* NMI	3.10	0.50	0.0001	0	-		
Not CGI and NMI	6.18	3.57	0.0001	3.08	0.0001	0	-
Not CGI and *not* NMI	5.41	2.81	0.0001	2.31	0.0001	-0.77	0.0001

TABLE C: Nucleosome occupancy scores from Schwartz et al. [1], grouped by the four genomic regions as shown in Figure 8a of the main text. Δ denotes the difference between mean scores, and *p*-values correspond to permutation tests for mean differences, applied due to non-normality of the data. Due to large sample sizes (see Table 2 in the main text), all of the mean differences are statistically significant.

Region	Mean score	CGI and NMI		CGI *not* NMI		*Not* CGI and NMI	
		Δ	*p*-value	Δ	*p*-value	Δ	*p*-value
CGI and NMI	134.03	0	-				
CGI *not* NMI	235.47	101.44	0.0001	0	-		
Not CGI and NMI	187.86	53.83	0.0001	-47.61	0.0001	0	-
Not CGI and *not* NMI	154.98	20.96	0.0001	-80.49	0.0001	-32.87	0.0001

TABLE D: Nucleosome occupancy scores from Yazdi et al. [2], grouped by the four genomic regions as shown in Figure 8b of the main text. See also the caption of Table C.

References

1. Schwartz U, Németh A, Diermeier S, Exler JH, Hansch S, Maldonado R, Heizinger L, Merkl R, Lāngst G. Characterizing the nuclease accessibility of DNA in human cells to map higher order structures of chromatin. Nucleic Acids Res. 2019; 47(3):1239–1254

2. Yazdi PG, Pedersen BA, Taylor JF, Khattab OS, Chen YH, Chen Y, Jacobsen SE, Wang PH. Nucleosome organization in human embryonic stem cells. PloS one. 2015; 10(8):e0136314.
